# Population Genetics of Overwintering Monarch Butterflies, *Danaus plexippus* (Linnaeus), from Central Mexico Inferred from Mitochondrial DNA and Microsatellite Markers

**DOI:** 10.1093/jhered/esw071

**Published:** 2016-12-21

**Authors:** Edward Pfeiler, Nestor O. Nazario-Yepiz, Fernan Pérez-Gálvez, Cristina Alejandra Chávez-Mora, Mariana Ramírez Loustalot Laclette, Eduardo Rendón-Salinas, Therese Ann Markow

**Affiliations:** 1 From the Centro de Investigación en Alimentación y Desarrollo, A.C., Unidad Guaymas, Guaymas, Sonora C.P. 85480, México (Pfeiler); Laboratorio Nacional de Genómica para la Biodiversidad (LANGEBIO), Centro de Investigación y de Estudios Avanzados del Instituto Politécnico Nacional (CINVESTAV), Irapuato, Guanajuato C.P. 36821, México (Nazario-Yepiz, Pérez-Gálvez, Chávez-Mora, Laclette, and Markow); World Wildlife Fund–Mexico, Programa Mariposa Monarca, Michoacán C.P. 61450, México (Rendón-Salinas); and Division of Biological Sciences, University of California, San Diego, La Jolla, CA 92093 (Markow).

**Keywords:** Bayesian skyline analysis, demographic history, effective population size, genetic diversity, haplotype networks, mismatch distribution, population structure

## Abstract

Population genetic variation and demographic history in *Danaus plexippus* (L.), from Mexico were assessed based on analyses of mitochondrial cytochrome *c* oxidase subunit I (COI; 658 bp) and subunit II (COII; 503 bp) gene segments and 7 microsatellite loci. The sample of 133 individuals included both migratory monarchs, mainly from 4 overwintering sites within the Monarch Butterfly Biosphere Reserve (MBBR) in central Mexico (states of Michoacán and México), and a nonmigratory population from Irapuato, Guanajuato. Haplotype (*h*) and nucleotide (*π*) diversities were relatively low, averaging 0.466 and 0.00073, respectively, for COI, and 0.629 and 0.00245 for COII. Analysis of molecular variance of the COI data set, which included additional GenBank sequences from a nonmigratory Costa Rican population, showed significant population structure between Mexican migratory monarchs and nonmigratory monarchs from both Mexico and Costa Rica, suggesting limited gene flow between the 2 behaviorally distinct groups. Interestingly, while the COI haplotype frequencies of the nonmigratory populations differed from the migratory, they were similar to each other, despite the great physical distance between them. Microsatellite analyses, however, suggested a lack of structure between the 2 groups, possibly owing to the number of significant deviations from Hardy–Weinberg equilibrium resulting from heterzoygote deficiencies found for most of the loci. Estimates of demographic history of the combined migratory MBBR monarch population, based on the mismatch distribution and Bayesian skyline analyses of the concatenated COI and COII data set (*n* = 89) suggested a population expansion dating to the late Pleistocene (~35000–40000 years before present) followed by a stable effective female population size (*N*_ef_) of about 6 million over the last 10000 years.

Monarch butterflies *Danaus plexippus* (L.) (Nymphalidae: Danainae) are best known for their impressively long distance annual migrations (up to ~4000 km) from their summer feeding and breeding ranges in the eastern USA and southern Canada to overwintering sites in the Monarch Butterfly Biosphere Reserve (MBBR) in the mountains of central Mexico (states of Michoacán and México), their huge numbers (tens to hundreds of millions) in the overwintering sites, and their return migration north in the spring after breaking reproductive diapause (Urquhart and Urquhart 1977, 1979). More than simply iconic and charismatic animals, the tight evolutionary linkage of monarchs to their milkweed host plants, *Asclepias* spp. (Apocynaceae), and their incredible migratory ability hold clues to basic biological processes observed in a wide range of organisms. Monarchs from western USA also undergo a shorter migration from the Rocky Mountain region to overwintering sites along the California coast (Urquhart and Urquhart 1979; Brower and Malcolm 1991). Hypothesized to have originated in the southern USA or northern Mexico, monarchs have dispersed widely, to Central America and northern South America and the Caribbean, and more recently across the Pacific and Atlantic Oceans (Pierce et al. 2014; Zhan et al. 2014). At the same time, however, there are North American breeding populations of monarchs that do not migrate. These nonmigratory populations occur in southern areas of the USA (Florida, Arizona, and California), Mexico, Central America, and islands of the Caribbean (Funk 1968; Urquhart et al. 1970; Dockx 2007; Morris et al. 2015; present study). Recently, increased ornamental planting of exotic tropical milkweed *Asclepias curassavica* L. in relatively warm regions of the southeastern USA has resulted in an increased number of monarchs breaking reproductive diapause during their autumn migration and becoming sedentary (i.e., nonmigratory) (Satterfield et al. 2015). In addition, some migratory monarchs from the eastern USA are now known to take an easterly route during the autumn, passing through southern Florida and Cuba, instead of Texas, on their way to Mexico (Dockx et al. 2004; Dockx 2007, 2012; Knight and Brower 2009).

The migratory population of *D. plexippus* from eastern North America has experienced large declines in recent years (Brower et al. 2012; Vidal and Rendón-Salinas 2014) and is listed in NatureServe and the Xerces Society (Jepson et al. 2015) as G4T1 (critically imperiled), although worldwide the species is listed as G4 (apparently secure); it has not yet been evaluated by the IUCN (IUCN 2015). Currently, the US Fish and Wildlife Service is reviewing a petition to list the *D. plexippus* as a threatened species under the Endangered Species Act. Factors underlying the decline of monarchs are complex. In addition to exposure to weather extremes throughout its range (e.g., Howard and Davis 2012), a major issue is their dependence upon milkweed species as the larval food plant. As milkweed disappears from North America owing to increased herbicide use in agricultural fields planted with genetically modified, herbicide-resistant crops, there are fewer resources for monarch reproduction (Pleasants and Oberhauser 2013). Another major issue is deforestation and forest degradation from illegal logging in adult overwintering sites (Vidal and Rendón-Salinas 2014; Vidal et al. 2014). In particular, Vidal and Rendón-Salinas (2014) documented significant declines in colony sizes and numbers of overwintering monarchs at localities both inside and outside the MBBR over a 10-year period.

Despite the enormous interest in this species, few basic population genetic data for monarchs from their overwintering sites in Mexico, useful for conservation efforts and programs, are available. A major factor in guiding management programs is the level of genetic diversity in a species and how that genetic diversity is structured in populations of that species (Frankham et al. 2010). Low levels of genetic diversity and low population size usually increase the risk of extinction. Thus, an estimate of effective population size using genetic methods is of critical importance (Hare et al. 2011). Also, if genetic diversity is low, but different variants exist in different parts of a species range, promotion of interbreeding among the populations can be critical to the survival of the species (Bonin et al. 2007; Schwartz et al. 2007).

Although hybridization among migratory and nonmigratory monarchs has been hypothesized (Dockx et al. 2004; Knight and Brower 2009), few molecular population genetic data are available that directly address this question. Knowing the extent of admixture among the 2 behaviorally different monarch populations in Florida and Cuba, as well as elsewhere in North America, would have important implications for captive breeding programs and conservation issues. The lack of (or limited) dispersal in nonmigratory populations, and potential differences in environmental extremes to which migrating butterflies are often exposed, could result in local differentiation or adaptation. For example, nonmigratory monarchs from South Florida, the Caribbean and Central America have smaller and often less elongated wings compared with the migratory populations from North America (Altizer and Davis 2010; Dockx 2012; Li et al. 2016) which suggest a genetic basis for these morphological traits and migratory status.

Previous studies utilizing a variety of genetic markers, including allozymes (Eanes and Koehn 1979; Shephard et al. 2002), mitochondrial DNA (mtDNA) (Brower and Boyce 1991; Brower and Jeansonne 2004), microsatellites (Lyons et al. 2012; Pierce et al. 2014) and whole genome analysis (Zhan et al. 2014) have consistently shown little genetic differentiation among migratory monarchs from widely separated geographic localities in North America, although only limited samples have been analyzed from Mexico overwintering sites, as mentioned above. Interestingly, the 2 most recent molecular genetic studies conducted on both migratory and nonmigratory monarchs give a conflicting picture of the extent of gene flow between the 2 populations. Whole genome analysis of monarchs from the USA and the overwintering sites in Mexico (migratory) compared with those from Belize and Costa Rica (nonmigratory) indicated significant population structure between the 2 populations (Zhan et al. 2014). Microsatellite data, however, suggest that the entire mainland North American populations of monarchs (excluding South Florida), including the migratory monarchs in USA and Mexico and the nonmigratory populations from Belize and Costa Rica, are panmictic (Pierce et al. 2014).

Extensive and critical gaps thus remain in our knowledge of the population genetics, genetic differentiation, and/or connectivity of monarch populations. Important questions to answer are: 1) What are the levels of genetic variability in monarchs from the major overwintering sites in the reserves and other sites in Mexico and elsewhere in North America? 2) To what degree are the migratory and nonmigratory populations genetically connected? 3) What can genetics tell us about their effective population sizes and historical demography? 4) Which genetic markers appear to be the most informative? We address these questions here by analyzing data obtained from direct sequencing of 658 base pairs (bp) of the barcode region of the mitochondrial cytochrome *c* oxidase subunit I gene (COI) and 503 bp of the mitochondrial *c* cytochrome oxidase subunit II gene (COII), together with analysis of a subset of the microsatellite loci developed for worldwide genetic studies in monarchs (Lyons et al. 2012; Pierce et al. 2014). We also included COI sequences of migratory and nonmigratory populations of *D. plexippus* available in GenBank and the Barcode of Life Data Systems (BOLD; Ratnasingham and Hebert 2007) in our analyses.

## Materials and methods

### Samples

Six subspecies of *D. plexippus* are currently recognized from mainland North America (including Central America), northern South America and the Caribbean, although 2 of these assignments are questionable (Warren et al. 2013). Because subspecies designations of *D. plexippus* are currently unstabilized and inconsistently used in the GenBank and BOLD databases, we omit subspecies names and instead use *D. plexippus* for the mainland population from North America (including Central America and the Caribbean), and then distinguish between “migratory” and “nonmigratory” populations. Although “nonmigratory” *D. plexippus* from Costa Rica are known to undergo short regional migrations (Haber 1993), they are isolated from the main migrating population from the MBBR.

**Figure 1.**
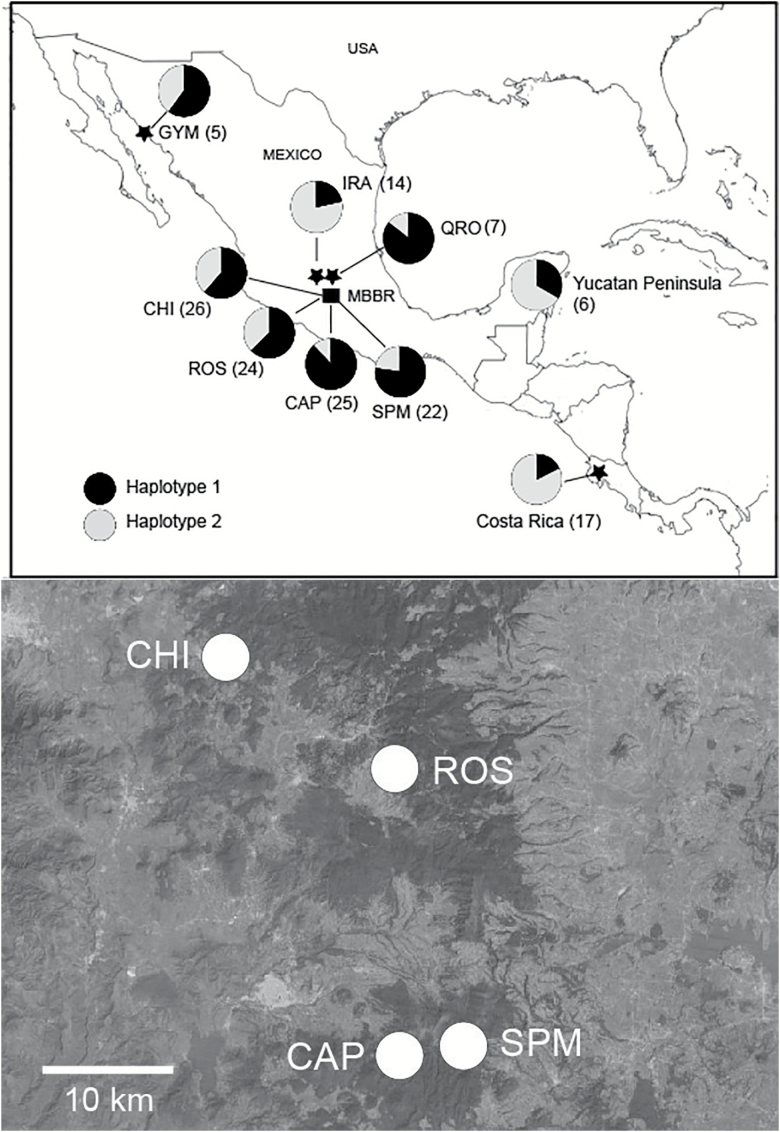
(Top) Map showing sampling sites for *Danaus plexippus* in Mexico and Costa Rica. Pie charts show relative frequencies of COI haplotypes 1 and 2 at each locality. Data for the Yucatán Peninsula and Costa Rica were taken from GenBank (Appendix Table A1). Numbers in parentheses are sample sizes for number of individuals showing COI haplotypes 1 or 2 only (total sample sizes for each mitochondrial gene and microsatellite locus analyzed are found in Tables 1 and 3, respectively). MBBR, Monarch Butterfly Biosphere Reserve; CHI, Sierra Chincua, Michoacán (19.6736°N, 100.3893°W); ROS, El Rosario, Michoacán (19.5959°N, 100.2634°W); SPM, San Pablo Malacatepec, Estado de México (19.4035°N, 100.2289°W); CAP, El Capulín, Estado de México (19.3894°N, 100.2706°W); QRO, Querétaro, Querétaro; GYM, Guaymas, Sonora; IRA, Irapuato, Guanajuato. The populations from Irapuato, Costa Rica, and the Yucatán Peninsula are nonmigratory; the remainder are migratory. (Bottom) Details of geographic locations and distances between sampling sites within the MBBR.

Four localities in the MBBR of central Mexico, 2 in Michoacán state (Sierra Chincua and El Rosario) and 2 in the state of Mexico (San Pablo Malacatepec and El Capulín) (Figure 1), were sampled for overwintering monarchs during February and March, 2014. Hand-held insect nets with extensions were used to collect aggregating butterflies from tree branches. Monarchs also were sampled from a resident population in Irapuato, Guanajuato, approximately 180 km NW of the MBBR, during August and September, 2015, and from an area outside of the Parque Nacional el Cimatario in Querétaro, Querétaro, approximately 130 km north of the MBBR, during November, 2015. The distance between Irapuato and Querétaro is about 100 km. The Irapuato population is nonmigratory and was sampled approximately 2–3 months before the arrival of monarchs at the MBBR, thus ensuring that no migratory individuals were included. The population from Querétaro was sampled during the migratory period in November, 2015. A small sample (*n* = 5) of migrating butterflies was also obtained on 31 October and 1 November, 2015 from a threatened coastal sand dune habitat near Guaymas, Sonora (Pfeiler et al. 2016). Butterflies were placed individually in glassine envelopes and stored at 4 °C. Sequence data for *D. plexippus* from additional sites in Mexico and other regions were also obtained from GenBank (Appendix Table A1) and BOLD (Ratnasingham and Hebert 2007).

### Mitochondrial DNA Amplification

Total genomic DNA was extracted from 2 legs of each butterfly using the DNeasy™ (QIAGEN Inc., Valencia, CA) protocol. The polymerase chain reaction (PCR) was used to amplify the 2 mitochondrial gene segments using primer pairs and standard assay conditions described previously for COI (Folmer et al. 1994) and COII (Liu and Beckenbach 1994). Forward and reverse sequencing reactions for both COI and COII were performed on an Applied Biosystems (Foster City, CA) ABI 3730XL DNA sequencer at the LANGEBIO core DNA sequencing facility using the PCR primers.

Sequences were proofread and aligned in either Clustal W (Thompson et al. 1994) or the MUSCLE algorithm (Edgar 2004) implemented in Aliview (Larsson 2014), followed by manual editing. The amplified COI fragment (658 bp), also known as the barcode segment (Ratnasingham and Hebert 2007), corresponds to nucleotide positions 1516–2173 in the complete mitochondrial genome of *D. plexippus* from the MBBR (GenBank KC836923; Servín-Garcidueñas and Martínez-Romero 2014). For the amplified COII gene, ambiguous or unresolved nucleotides were removed, resulting in a 503 bp segment corresponding to nucleotide positions 3156–3658 in the mitochondrial genome of *D. plexippus*. All alignments for both gene segments were straightforward, but owing to the generally low genetic diversity present, especially in the COI segment, original electropherograms were screened visually to check for possible reading errors. Electorpherograms for publicly available GenBank sequences of *D. plexippus* from Costa Rica (Janzen and Hallwachs 2009) and the Yucatán Peninsula (Prado et al. 2011) examined here are found in BOLD (Ratnasingham and Hebert 2007). Several nucleotide reading errors in output files, which would have resulted in assigning false haplotypes, were found and corrected in both GenBank records and our new sequences.

Translation of COI and COII sequences in MEGA version 5.0.5 (Tamura et al. 2011) revealed no frameshifts or stop codons. Little variation in base composition was found for both genes. Mean CG content was 29.0% (*n* = 125) in COI and 22.0% (*n* = 123) in COII. Together these results indicate that the barcode sequences analyzed represent mtDNA, and are not nuclear mitochondrial pseudogenes (numts).

### Microsatellite Amplification and Genotyping

The DNA extract described above was used to amplify 7 of the polymorphic microsatellite markers developed by Lyons et al. (2012) for *D. plexippus*. The selected loci included 5 dinucleotide repeats (loci 320, 208, 983, 223, and 854) and 2 trinucleotide repeats (loci 1679 and 165). In the MBBR sample (*n* = 27) of Pierce et al. (2014), 4 of these loci (320, 208, 1679, and 854) were in Hardy–Weinberg equilibrium (HWE), and 2 (983 and 165) were not in HWE; locus 223 was not included in their study. We chose these particular loci to assess whether any changes in HWE were apparent in MBBR samples taken in different years (Waples 2015); locus 223 has not been previously analyzed in monarchs from this region. In the 6-year interval (2008–2014) between sampling in Pierce et al. (2014) and the present study, estimated colony size of overwintering monarchs in the MBBR showed a drastic reduction of ~80% (Vidal and Rendón-Salinas 2014).

Primer sequences for each locus are given in Lyons et al. (2012). Forward primers were fluorescent labeled and PCR reactions were set up in 2 multiplex arrays. PCRs were carried out with a Type-it Microsatellite PCR Kit (QIAGEN) using primer concentrations of 0.2 µM and 20–50 ng of template DNA. After an initial incubation at 95 °C for 5 min, the multiplexed reaction was run 28 times through a temperature profile of 95 °C for 30 s, annealing temperatures of 60 °C or 64 °C (loci 854 and 165) for 90 s, 72 °C for 30 s, followed by a 20 min extension at 60 °C. Hi-Di™ Formamide was added to the PCR reactions to obtain the recommended dilution. Samples were analyzed in an Applied Biosystem 3730XL capillary sequencer with Dye Set G5 and GeneScan™ 500 LIZ™ dye size standard.

Microsatellite genotypes were analyzed with the microsatellite plugin of Geneious software (Biomatters Limited) and Microsatellite Analyzer (MSA) version 4.05 (Dieringer and Schlötterer 2003). Observed and expected heterozygosities at each locus for each population were calculated in ARLEQUIN version 3.5.2.2 (Excofﬁer and Lischer 2010). Deviations from HWE were tested in ARLEQUIN using a Markov chain approximation. All estimates were assessed for significance using a test analogous to Fisher’s exact test, with 100000 steps in the Markov chain and 10000 dememorization steps.

### Genetic Diversity

Calculations of genetic diversity indices of COI, COII and the concatenated data set for MBBR samples were performed in DnaSP version 5.10.01 (Librado and Rozas 2009). Neutrality tests, Tajima’s (1989)*D* and Fu’s (1997)*F*_S_, were carried out in ARLEQUIN. We analyzed COI and COII separately, as well as concatenated, in order to compare our results on COI with sequences of other nonmigratory populations from North America mined from GenBank and BOLD in which corresponding COII sequences were not available. The concatenated data set was used for tests of historical demography of migratory monarchs from the MBBR described below.

Hierarchical analysis of molecular variance (AMOVA; Excofﬁer et al. 1992) performed in ARLEQUIN using the COI data set was used to test for population structure in *D. plexippus* populations from Mexico and Costa Rica. For this analysis, we assumed that each of the 4 MBBR collection sites represented separate populations. The Guaymas population was omitted owing to small sample size (*n* = 5). In addition, only the 19 complete (656–658 bp) barcode sequences from Costa Rica listed in the Appendix Table A1 were analyzed. Populations were divided into 2 groups representing the migratory (MBBR and Querétaro) and nonmigratory (Irapuato and Costa Rica) populations. The hierarchical AMOVA partitioned genetic variation among localities relative to the total sample (*Φ*_ST_), among localities within groups (*Φ*_SC_), and among the migratory and nonmigratory groups (*Φ*_CT_). The calculation of signiﬁcance of the ﬁxation indices *Φ*_ST_, *Φ*_SC_, and *Φ*_CT_ (α = 0.05) was based on 10000 permutations of the data matrix. Pairwise estimates of *Φ*_ST_ among populations, and estimates of number of migrants per generation (*N*_m_) among populations assumed to be in mutation-drift equilibrium (Slatkin 1991), were also calculated in ARLEQUIN. In addition, we conducted separate AMOVAs on 1) the COII data set for *D. plexippus* from Mexico, 2) the concatenated COI and COII data set from the 4 MBBR sites, and 3) the microsatellite data set from Mexico (excluding Guaymas).

Haplotype networks of both COI and COII sequences were constructed using statistical parsimony implemented in TCS version 1.21 (Clement et al. 2000). The connection limit among haplotypes was set to the default value of 95%.

### Demographic History

For analyses of demographic history we used the concatenated COI and COII data set from *D. plexippus* from the MBBR (1161 bp; *n* = 89) which were collected during a single overwintering period. Changes in effective female population size (*N*_ef_) over time in monarchs from the MBBR were estimated with 1) analysis of the distribution of pairwise differences among haplotypes (mismatch distribution) (Rogers and Harpending 1992; Harpending 1994) performed in ARLEQUIN and 2) Bayesian skyline analysis (Drummond et al. 2005). For the mismatch distribution, the signiﬁcance of the estimated parameters of the sudden expansion model was obtained from the sum of square deviations (*SSD*) statistic and the raggedness statistic (*rg*), and their corresponding *P* values. The sudden expansion model is rejected at *P* < 0.05.

Bayesian Skyline analysis utilizes Markov chain Monte Carlo (MCMC) sampling of sequence data to estimate a posterior distribution of effective population size through time (Drummond et al. 2005). The model of nucleotide substitution that best ﬁt the concatenated data set, determined with Modeltest, version 3.7 (Posada and Crandall 1998) using the Akaike information criterion, was TrN (Tamura and Nei 1993). The Bayesian skyline analysis was run using the HKY substitution model (very similar to the TrN model [Yang 1994] and the best alternative available) in BEAUti and BEAST versions 1.8.2 (Drummond et al. 2012). Five million iterations of the MCMC chains were run and sampled every 1000 iterations. The Bayesian skyline plots were generated with TRACER version 1.6 (Rambaut et al. 2014). We assumed a neutral mutation rate per site per generation (*μ*) of 2.9 × 10^−9^ based on an estimated 2.3% pairwise sequence divergence rate per million years for mtDNA (Brower 1994) and 4 generations per year (Brower 1995).

### Data Availability

All unique haplotypes of the new COI and COII sequences reported here are available in GenBank (accession numbers KX110357–KX110382). Microsatellite genotypes have been deposited in Dryad: http://dx.doi.org/10.5061/dryad.64m4n (Baker 2013).

## Results

### Genetic Diversity

**Figure 2.**
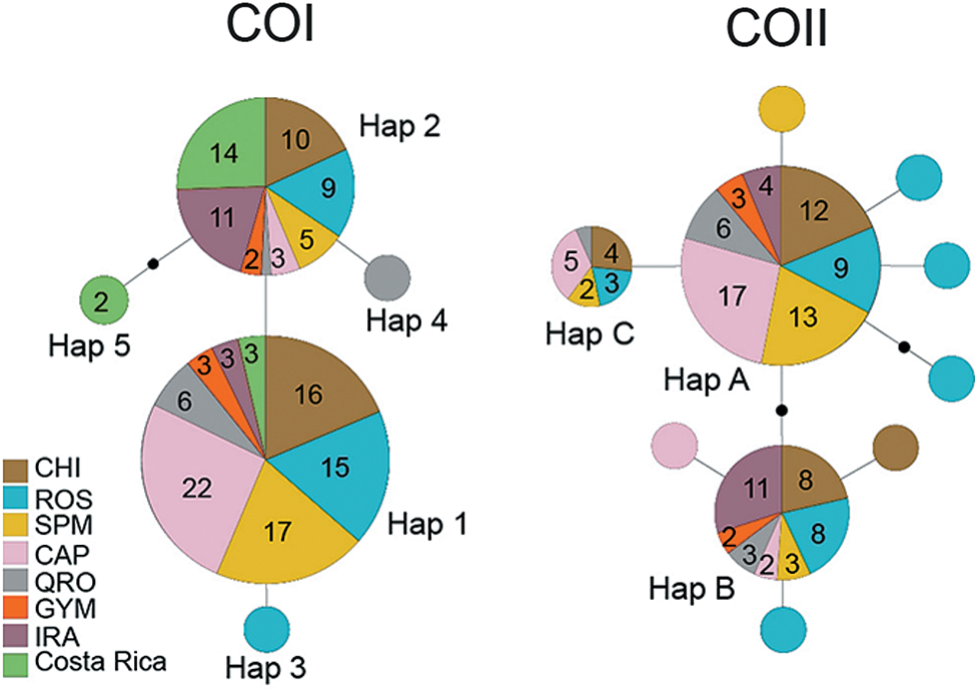
TCS haplotype networks for the COI (658 bp; *n* = 144) and COII (503 bp; *n* = 123) data sets in *Danaus plexippus* from Mexico and Costa Rica (see legend of Figure 1 for locality abbreviations and migratory and nonmigratory populations). Each line segment between haplotypes represents a single mutation. Inferred intermediate haplotypes that were not sampled are shown as black dots. Size of the circles is scaled approximately to haplotype frequency. Numbers within circles represent number of individuals with that haplotype, if greater than 1. The 7 singleton haplotypes of COII are not labeled.

Haplotype diversity (*h*) and nucleotide diversity (*π*) in the COI gene segment were generally low in each of the 7 populations sampled from Mexico, with overall values of *h* = 0.466 and *π* = 0.00073 in the combined sample (*n* = 125; Table 1). Only 3 variable sites and 4 haplotypes were found in the combined sample. Two of the 4 haplotypes were singletons (haplotypes 3 and 4) consisting of a third codon position transition in an individual from El Rosario and one from Querétaro, respectively (Figure 2). The common haplotype (haplotype 1) was found in 82 individuals from Mexico (66% of the sample), with the second most common haplotype (haplotype 2) being present in the remaining 41 individuals (33%) (Figure 2). Similarly, low genetic diversity was seen in monarchs from Costa Rica (*n* = 19; Table 1), in which 2 individuals also possessed a new haplotype consisting of 2 nucleotide substitutions (haplotype 5) not seen in Mexico (Figure 2). Of the complete (656–658 bp) COI barcode sequences for 155 *D. plexippus* (125 from Mexico [Table 1] and 30 from GenBank [Appendix Table A1]), only 6 variable sites and 6 haplotypes were present, again reflecting the low genetic variability in this gene (*h* = 0.505 ± 0.024; *π =* 0.00087 ± 0.00007). Three of the 6 haplotypes (haplotypes 3, 4, and an additional haplotype 6 [see below]) were singletons each consisting of a single nucleotide substitution. All nucleotide substitutions were synonymous third codon position transitions.

**Table 1.** Summary of genetic diversity indices and results of neutrality tests (Tajima’s *D* and Fu’s *F*_S_) in the COI (658 bp) and COII (503 bp) gene segments in *Danaus plexippus* from 7 localities in Mexico and 1 site in Costa Rica (COI only)

Locality/abbreviation	*n*	*k*	*K*	*h* (±SD)	*π* (±SD)	*D*	*F*_S_
**COI**							
MEXICO							
Sierra Chincua (CHI)	26	1	2	0.492 ± 0.051	0.00075 ± 0.00008	1.437	1.523
El Rosario (ROS)	25	2	3	0.530 ± 0.064	0.00085 ± 0.00013	0.124	0.164
San Pablo Malacatepec (SPM)	22	1	2	0.356 ± 0.100	0.00054 ± 0.00015	0.593	0.911
El Capulín (CAP)	25	1	2	0.220 ± 0.100	0.00033 ± 0.00015	−0.281	0.195
Querétaro (QRO)	8	2	3	0.464 ± 0.200	0.00103 ± 0.00048	−0.448	−0.478
Irapuato (IRA)	14	1	2	0.363 ± 0.130	0.00055 ± 0.00020	0.324	0.643
Guaymas (GYM)	5	1	2	0.600 ± 0.175	0.00091 ± 0.00027	1.225	0.626
Total Mexico **COI**	125	3	4	0.466 ± 0.030	0.00073 ± 0.00006	−0.229	−0.359
COSTA RICA	19	3	3	0.444 ± 0.130	0.00104 ± 0.00036	−0.570	0.338
**COII**							
MEXICO							
Sierra Chincua (CHI)	25	4	4	0.667 ± 0.059	0.00262 ± 0.00032	0.665	0.923
El Rosario (ROS)	24	8	7	0.757 ± 0.058	0.00323 ± 0.00040	−0.777	−1.495
San Pablo Malacatepec (SPM)	19	4	4	0.520 ± 0.123	0.00172 ± 0.00049	−0.718	−0.392
El Capulín (CAP)	25	4	4	0.510 ± 0.102	0.00170 ± 0.00049	−0.525	−0.150
Queretaro (QRO)	10	3	3	0.600 ± 0.131	0.00225 ± 0.00056	0.247	0.723
Irapuato (IRA)	15	2	2	0.419 ± 0.113	0.00167 ± 0.00045	0.954	2.222
Guaymas (GYM)	5	2	2	0.600 ± 0.175	0.00239 ± 0.00070	1.459	1.688
Total Mexico **COII**	123	11	10	0.629 ± 0.031	0.00245 ± 0.00016	−1.008	−2.629
Concatenated **COI** + **COII** MBBR	89	13	12	0.675 ± 0.040	0.00144 ± 0.00013	−0.953	−3.321

*n*, number of individuals; *k*, number of variable sites; *K*, number of haplotypes; *h*, haplotype diversity; *π*, nucleotide diversity. MBBR = combined populations CHI, ROS, SPM, and CAP. None of the values for *D* and *F*_S_ were significant at the 0.05 level. Nonmigratory populations are highlighted in gray.

A comparison of the frequencies of COI haplotypes 1 and 2 for all samples analyzed from Mexico and Costa Rica is shown in Figure 1. The nonmigratory Costa Rica samples were from the Area de Conservación Guanacaste (ACG), Guanacaste Province in northwestern Costa Rica (Janzen and Hallwachs 2009), approximately 1800 km SE of the MBBR. We also included GenBank data from a small sample (*n* = 7) of nonmigratory monarchs from 7 sites on the Yucatán Peninsula, Mexico (states of Yucatán, Campeche, and Quintana Roo [Appendix Table A1]) located about 1200 km east of the MBBR (Prado et al. 2011). Because several of these peninsular sites were separated by distances of approximately 250–300 km, and owing to the fact that only 3 sequences were complete (658 bp) barcodes (Appendix Table A1), these data were not incorporated in the other genetic analyses reported here. In addition to possessing both haplotypes 1 and 2, 1 complete barcode from Quintana Roo (GU659711) possessed a distinct haplotype (haplotype 6; Appendix Table A1) not seen in the other samples analyzed. Figure 1 shows that frequencies of COI haplotypes 1 and 2 in migratory and nonmigratory monarch populations showed clear differences. The mean frequency of haplotype 1 in the migratory populations from Mexico (MBBR, Querétaro, and Guaymas; *n* = 109) was 74% but was reduced to approximately 22% in the nonmigratory populations from Costa Rica, Irapuato, Mexico, and the Yucatán Peninsula, Mexico (*n* = 37).

Genetic diversity in the COII gene segment of the combined sample from Mexico (*h* = 0.629; *π* = 0.00245; *n* = 123) was higher than that seen in COI, and was comprised of 11 variable sites and 10 haplotypes (Table 1; Fig. 2). The common COII haplotype (haplotype A) was present in 64 individuals (52% of the sample); haplotypes B and C were present in 37 and 15 individuals, (30% and 12%), respectively. The remaining 7 haplotypes were singletons. Electropherograms were carefully checked, and no reading errors were found in the singletons, which included 4 haplotypes with third codon position transitions, one with a first position transition, one with a third position transversion (synonymous) and one haplotype consisting of 2 first position nonsynonymous transitions. GenBank data for the corresponding 503 bp COII segment reported previously for 5 individuals of migratory monarchs from Michoacán, Mexico (Brower and Jeansonne 2004) showed only haplotype A (AY569145–48) and haplotype B (AY569149). The predominant COII haplotype in the nonmigratory Irapuato population was haplotype B (11 of 15 individuals), with haplotype A found in the 4 remaining individuals. Thus, shifts in predominant allele frequencies in nonmigratory populations, similar to that seen in COI, also appear to be present in COII, although more data from nonmigratory populations will be required for confirmation.

Genetic diversity of the concatenated COI and COII data set (*h* = 0.675; *π* = 0.00144; *n* = 89) reflected the higher diversity values present in COII. Neutrality tests were not significant for the separate or concatenated gene segments (Table 1).

Variation in the 7 microsatellite loci over all populations of *D. plexippus* from Mexico is shown in Table 2. Observed heterozygosity ranged from 0.308 to 0.857. The maximum number of alleles ranged from 4 to 17. Except for locus 165, all loci showed significant deviations from HWE. These results are in contrast to those obtained by Pierce et al. (2014) for migratory monarchs in which their combined sample from the USA (*n* = 200) and their sample from Mexico (*n* = 27) each showed significant deviation from HWE at locus 165. Overall, however, 8 of the 11 loci analyzed in detail by Pierce et al. (2014) were not in HWE, a pattern similar to what we found.

**Table 2.** Summary of results for each microsatellite locus averaged over 7 populations of *Danaus plexippus* from Mexico

Locus	*n*	*H*_obs_	*H*_exp_	*P*	Allele size (bp)	No. alleles	Repeat motif
320	130	0.531	0.867^a^	<0.000	156–188	16	at
208	133	0.857	0.838^a^	<0.000	180–222	17	at
1679	133	0.421	0.592^a^	<0.000	216–225	4	tat
983	133	0.534	0.709^a^	<0.000	230–256	11	ac
223	133	0.617	0.836^a^	<0.000	190–228	17	tg
854	133	0.308	0.458^a^	<0.000	234–254	5	at
165	133	0.481	0.499	0.903	198–210	5	tat

*n*, number of individuals genotyped; H_obs_ and H_exp_, observed and expected heterozygosities, and the corresponding *P* values.

a Significant deviation from Hardy–Weinberg equilibrium.

Microsatellite analyses of the 7 loci in each of our 7 populations from Mexico, together with results of Pierce et al. (2014) on overwintering monarchs from the MBBR for comparison, are shown in Table 3. Consistent with results from combined populations (Table 2), many loci showed heterozygote deficiencies and significant deviations from HWE when populations were analyzed separately. Also consistent with results shown in Table 2, locus 165 was in HWE in all populations, except in the nonmigratory population from Irapuato and the migratory population from the MBBR studied by Pierce et al. (2014). Locus 983 was in HWE in 3 of the 4 populations we sampled from the MBBR, and in the combined MBBR sample, but was not in HWE in the MBBR sample of Pierce et al. (2014). Inconsistent results of HWE analysis on migratory monarchs sampled from the same region in Mexico in the 2 different studies raise doubts about the utility of microsatellite markers to understand subtle differences in population genetic structure in this species, a point that we address in more detail in the Discussion.

**Table 3.** Variation at seven microsatellite loci for each population of *Danaus plexippus* from Mexico collected in 2014 and 2015 together with results from Pierce et al. (2014) for specimens collected in 2008.

Pop/year	*n*	Locus	320	208	1679	983	223	854	165
CHI 2014	26	H_obs_	**0.417**	0.885	**0.423**	0.577	0.654	0.385	0.577
		H_exp_	**0.834** ^a^	0,801	**0.681** ^a^	0.691	0.811	0.486	0.497
ROS 2014	26	H_obs_	**0.560**	0.923	0.654	0.692	0.692	0.308	0.538
		H_exp_	**0.857** ^a^	0.841	0.594	0.590	0.819	0.413	0.542
SPM 2014	26	H_obs_	0.577	0.654	**0.269**	0.692	**0.423**	**0.269**	0.615
		H_exp_	0.856	0.762	**0.613** ^a^	0.839	**0.831** ^a^	**0.572** ^a^	0.521
CAP 2014	25	H_obs_	**0.280**	0.880	0.440	**0.520**	**0.680**	0.400	0.440
		H_exp_	**0.847** ^a^	0.844	0.478	**0.633** ^a^	**0.829** ^a^	0.483	0.478
Total MBBR	103	H_obs_	**0.460**	0.835	**0.447**	0.621	**0.612**	**0.340**	0.544
2014		H_exp_	**0.861** ^a^	**0.819** ^a^	**0.600** ^a^	0.698	**0.836** ^a^	**0.490** ^a^	0.504
Pierce et al.	27	H_obs_	0.778	0.519	0.519	**0.407**	NA	0.462	**0.192**
MBBR 2008		H_exp_	0.860	0.784	0.638	**0.806** ^a^	NA	0.609	**0.438** ^a^
QRO 2015	10	H_obs_	**0.600**	0.900	0.400	**0.300**	0.700	0.200	0.400
		H_exp_	**0.921** ^a^	0.926	0.505	**0.695** ^a^	0.921	0.432	0.574
IRA 2015	15	H_obs_	0.867	0.933	**0.133**	**0.267**	**0.600**	0.200	**0.133**
		H_exp_	0.818	0.871	**0.549** ^a^	**0.747** ^a^	**0.733** ^a^	0.301	**0.432** ^a^

Results from the MBBR are shaded in pink; the nonmigratory Irapuato population is shaded in gray.

a Significant deviation from HWE after a sequential Bonferroni correction for multiple comparisons (Rice, 1989) is shown in bold type. NA, not analyzed.

### Genetic Structure

The hierarchical AMOVA of the combined migratory and nonmigratory COI data set of *D. plexippus* from Mexico and Costa Rica showed significant population structure (overall *Φ*_ST_ = 0.396; *P* < 0.000), with 38.61% of the genetic variation distributed between migratory and nonmigratory groups (*Φ*_CT_ = 0.386; *P* = 0.047) (Table 4). Less than 1% of the genetic variation was distributed among populations within the 2 groups (*Φ*_SC_ = 0.015; *P* = 0.240). The remaining 60% of the genetic variation was found within populations.

**Table 4.** Hierarchical AMOVA of the COI data set in *Danaus plexippus* grouped by migratory (MBBR and Querétaro, Mexico; *n* = 106) versus nonmigratory (Irapuato, Mexico, and Costa Rica; *n* = 33)

Source of variation	*df*	Sum of squares	Variance components	% of variation
Among groups	1	7.653	0.14619 Va	38.61
Among populations within groups	5	1.499	0.00359 Vb	0.95
Within populations	132	30.215	0.22890 Vc	60.45
Total	138	39.367	0.37868	100.01
Fixation indices				
*Φ*_ST_ = 0.396^a^ (*P* < 0.000)				
*Φ*_SC_ = 0.015 (*P* = 0.240)				
*Φ*_CT_ = 0.386^a^ (*P* = 0.047)				

a Significant at the 0.05 level.

Pairwise comparisons of *Φ*_ST_ suggested no significant structure among the migratory populations in Mexico, or between the 2 widely separated nonmigratory populations in Irapuato, Mexico, and Costa Rica (Table 5). All pairwise comparisons of *Φ*_ST_ between Costa Rica and the migratory populations were significant after applying a Bonferroni correction for multiple comparisons. However, only 2 (SPM and CAP) of the 5 values of *Φ*_ST_ for Irapuato and the migratory populations in Mexico were significant. Nonetheless, the 3 nonsignificant values of *Φ*_ST_ in Irapuato were relatively high and similar to those seen in Costa Rica. In addition, *P* values of the 3 *Φ*_ST_ values were < 0.05. All estimates of *N*_m_ (0.32–1.64 migrants per generation) between migratory and both nonmigratory populations were low (Table 5), suggesting little gene flow between the two.

**Table 5.** Pairwise comparisons of *Φ*_ST_ (below the diagonal) and number of migrants per generation (*N*_m_; above the diagonal) among the 6 populations of *Danaus plexippus* from central Mexico (see legend of Figure 1 for locality abbreviations), and 1 population from Costa Rica, based on COI sequences

	CHI (26)	ROS (25)	SPM (22)	CAP (25)	QRO (8)	IRA (14)	Costa Rica (19)
CHI	**—**	inf	33.60	3.21	inf	1.64	1.39
ROS	–0.036	**—**	inf	4.47	inf	1.58	1.34
SPM	0.015	–0.002	**—**	inf	inf	0.63	0.68
CAP	0.135	0.101	–0.003	**—**	27.74	0.32	0.40
QRO	–0.019	–0.034	–0.052	0.018	**—**	0.92	1.03
IRA	0.233	0.241	**0.443** ^a^	**0.613** ^a^	0.351	**—**	inf
Costa Rica	**0.265** ^a^	**0.272** ^a^	**0.423** ^a^	**0.557** ^a^	**0.326** ^a^	–0.016	**—**

Number of individuals from each population shown in parentheses. Nonmigratory populations are highlighted in gray.

a Statistically significant *Φ*_ST_ values after a sequential Bonferonni correction are shown in bold type. Abbreviation “inf” indicates value for *N*_m_ is undefined and approaches panmixia.

The AMOVA of the COII data set, which included 5 migratory populations and one nonmigratory population (Irapuato) from central Mexico, indicated that 10% of the genetic variation occurred among populations and that significant structure was present (overall *Φ*_ST_ = 0.102; *P* = 0.002). Pairwise comparisons of *Φ*_ST_ again suggested no significant structure among the migratory populations in Mexico (Table 6). The nonmigratory Irapuato population showed significant structure compared with the SPM and CAP migratory populations, but no structure was found when compared with the other 3 migratory populations, the same result as seen with COI (Table 5). The *P* values for these 3 comparisons were also <0.05.

**Table 6.** Pairwise comparisons of *Φ*_ST_ (below the diagonal) and number of migrants per generation (*N*_m_; above the diagonal) among 6 populations of *Danaus plexippus* from central Mexico based on COII sequences

	CHI (25)	ROS (24)	SPM (19)	CAP (25)	QRO (10)	IRA (15)
CHI	**—**	inf	15.23	7.10	inf	2.33
ROS	–0.034	**—**	14.26	6.42	inf	3.17
SPM	0.032	0.034	**—**	inf	inf	0.68
CAP	0.066	0.072	–0.028	**—**	41.06	0.57
QRO	–0.064	–0.060	–0.032	0.012	**—**	1.56
IRA	0.177	0.136	**0.423** ^a^	**0.469** ^a^	0.243	**—**

Nonmigratory populations are highlighted in gray.

a Statistically significant *Φ*_ST_ values after a sequential Bonferroni correction are shown in bold type.

The AMOVA of the concatenated data set from the 4 MBBR sites indicated that most (97%) of the genetic variation occurred within populations and that the overall *Φ*_ST_ (0.026) was not significant (*P* = 0.15). None of the pairwise comparisons of *Φ*_ST_ among the 4 MBBR sites was significant (not shown).

In contrast to the mtDNA results, but in agreement with Pierce et al. (2014), microsatellite analysis revealed that none of the pairwise comparisons of *F*_ST_ between the 6 populations of *D. plexippus* from Mexico, including the nonmigratory Irapuato population, were significant (Table 7), indicating a lack of population structure. The *P* value, however, for the comparison of *F*_ST_ in the Irapuato population and the migratory El Rosario population (*P* = 0.0039), however, was only slightly higher than the cutoff for significance using the Bonferroni correction (*P* = 0.0033). An analysis of the discrepancies in results of population structure between mtDNA and microsatellite markers is provided in the Discussion.

**Table 7.** Pairwise comparisons of *F*_ST_ (below the diagonal) and number of migrants per generation (*N*_m_; above the diagonal) among 6 populations of *Danaus plexippus* from Mexico based on analysis of 7 microsatellite loci

	CHI (26)	ROS (26)	SPM (26)	CAP (25)	QRO (10)	IRA (15)
CHI	**—**	11.38	inf	22.12	68.15	11.40
ROS	0.021	**—**	10.13	inf	28.89	5.90
SPM	−0.001	0.024	**—**	14.29	64.57	10.07
CAP	0.011	−0.000	0.017	**—**	29.02	14.98
QRO	0.004	0.009	0.004	0.009	**—**	9.61
IRA	0.021	0.041	0.024	0.016	0.025	**—**

None of the comparisons of *F*_ST_ were significant. Nonmigratory populations are highlighted in gray.

### Demographic History

**Figure 3.**
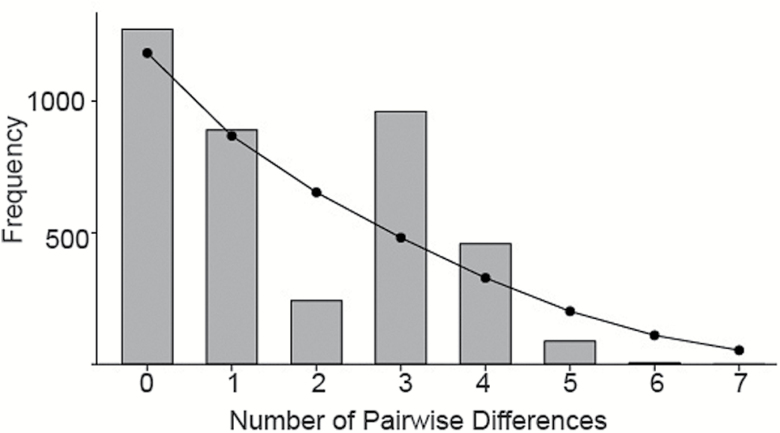
Demographic history of *Danaus plexippus* from the MBBR estimated from the mismatch distribution of the concatenated COI and COII data set (1161 bp; *n* = 89). Vertical bars show the observed distribution of pairwise differences among haplotypes, with the solid line representing the expected distribution under the sudden expansion model.

A plot of the distribution of pairwise differences among haplotypes in the concatenated data set (Figure 3) showed relatively good agreement with the expected distribution for populations that have undergone expansions (Harpending 1994). The mismatch distribution test statistics *SSD* (0.029; *P* = 0.35) and *rg* (0.096; *P* = 0.33) were small and not statistically signiﬁcant at the 0.05 level indicating that the sudden expansion model could not be rejected. The value found for *τ*, the time to the population expansion, where *τ* = 2*ut*, and *u* is the mutation rate for the entire gene segment, and *t* is the number of generations since the expansion (Rogers and Harpending 1992), was 3.695 (95% confidence intervals: 0.543, 6.594). Assuming 2.3% pairwise divergence per million years (Brower 1994), the mean mutation rate per site per generation in the 1161 bp segment for a single lineage is (1161) × (1.15 × 10^−8^) or 1.335 × 10^−5^. Based on these values, and assuming 4 generations per year, the estimated time to the population expansion in *D. plexippus* (with 95% confidence intervals) was 34597 (5084–61742) years ago.

**Figure 4.**
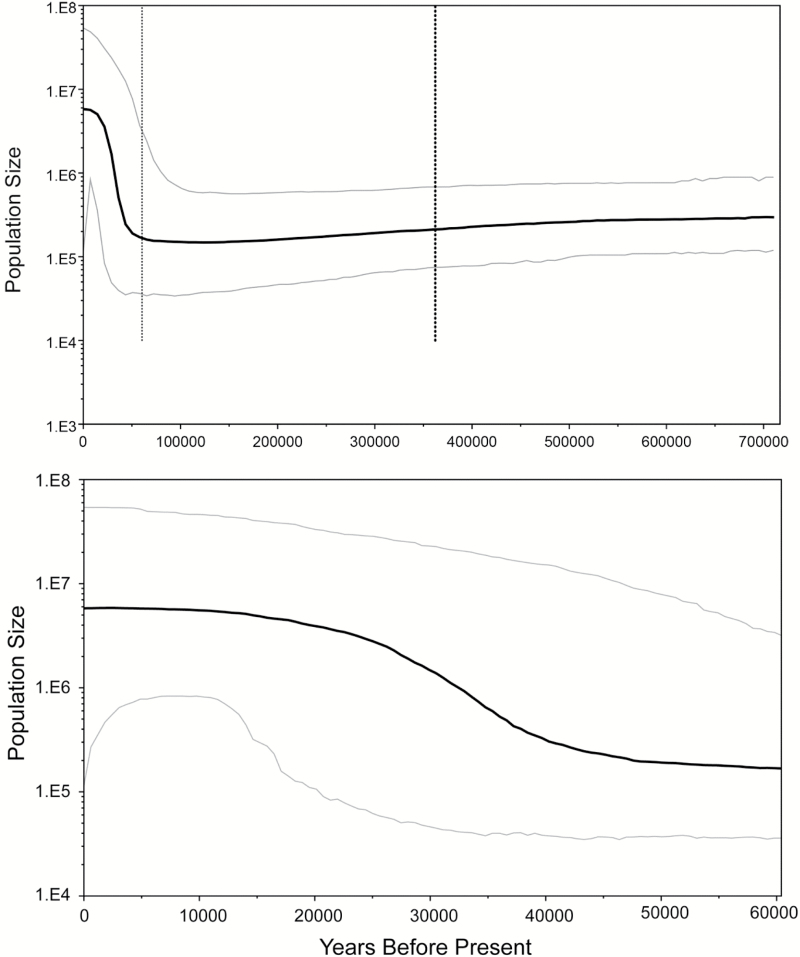
(Top) Bayesian skyline plot (BSP) showing the demographic history of *Danaus plexippus* from the MBBR over approximately the last 700000 years inferred from the concatenated COI and COII data set. The estimated changes in effective female population size (*N*_ef_) over time is given on a logarithmic scale. The middle solid line represents the median estimate of population size; the upper and lower lines represent the 95% HPD (highest posterior density) intervals. The vertical dotted lines represent the median estimate (right) and lower 95% HPD (left) of time to the most recent common ancestor. (Bottom) Expanded portion of the BSP showing changes in *N*_ef_ over the last 60000 years.

Bayesian skyline analysis (Figure 4) showed a large increase in female effective population size (*N*_ef_) in *D. plexippus* beginning about 40000 years before present, which then remained stable over the last 10000 years at a median estimate of about 6 million individuals. The estimated date of the beginning of the population expansion agrees remarkably well with that obtained from the mismatch distribution. Although large negative *F*_S_ values are also typically found in expanding populations (Fu 1997), the negative value shown for the concatenated data set (*F*_S_ = –3.321; Table 1) was not significant (*P* = 0.09).

## Discussion

Our mtDNA and microsatellite data suggest a large panmictic population of *D. plexippus* across the overwintering sites in central Mexico, which is consistent with other genetic studies utilizing a variety of markers showing panmixia in the entire North America migratory population of monarchs (Brower and Boyce 1991; Brower and Jeansonne 2004; Pierce et al. 2014; Zhan et al. 2014). The overwintering sites contain individuals that were produced over a wide geographic area of the north prior to their arrival in the reserves (Miller et al. 2012), and therefore it is impossible to know the mating histories of the migrating parents of the monarchs sampled in the reserves. The panmixia therefore likely reflects mating events during the spring and summer reproductive phase of the migratory population in USA and Canada.

Haplotype and nucleotide diversities were low in the COI gene segment in *D. plexippus*, and only slightly higher in the COII segment, in agreement with 2 previous mtDNA studies. Restriction fragment–length polymorphism analysis (Brower and Boyce 1991) and direct sequencing of an approximately 1500 bp segment of the COI and COII genes (Brower and Jeansonne 2004) showed very little genetic differentiation among monarchs from the USA (including eastern and western populations) and Mexico (MBBR), as well among a few individuals from northern South America and the Caribbean, but sample sizes from Michoacán, Mexico were low (≤12 individuals). Allozyme studies (Eanes and Koehn 1979; Shephard et al. 2002) also have indicated low levels of genetic variability in *D. plexippus* in general as well as little differentiation among the populations sampled from across large geographic areas of North America. In addition, microsatellite studies have shown a lack of genetic differentiation between eastern and western USA populations (Lyons et al. 2012).

Although genetic diversity is low, we show here that mtDNA sequence data from the first large sample of *D. plexippus* from Mexico can provide important insights into their population genetic structure and demographic history. We find it remarkable that although the overwintering sites of the eastern North America population of migratory monarchs have been known to the scientific community for 4 decades (Urquhart and Urquhart 1977, 1979), there have been so few DNA studies focusing on population genetics of overwintering individuals in the MBBR. In addition, the presence of monarchs that reside throughout the year in many of the states in central and southern Mexico (Vargas et al. 1996; Vidal and Rendón-Salinas 2014) provides a unique opportunity to examine in detail the extent of gene flow among migratory and nonmigratory populations, a topic that has not been adequately explored.

Two recent in-depth studies of monarch populations worldwide, one utilizing microsatellite data (Pierce et al. 2014) and the other whole genome analysis (Zhan et al. 2014), arrived at different conclusions concerning structure within North America populations. The microsatellite study concluded that populations from USA (with the exception of nonmigratory populations from South Florida and Puerto Rico), Mexico, Belize, and Costa Rica were panmictic, whereas the whole genome study concluded that the nonmigratory populations from Costa Rica and Belize showed genetic structure with respect to migratory populations from Mexico and USA. In both studies, the monarchs sampled from Mexico were migratory and obtained from the overwintering sites. Our mtDNA analyses agree with results of Zhan et al. (2014) in showing structure among the 2 behaviorally distinct populations, although the evidence for structure is somewhat stronger for the Costa Rica population. The nonmigratory Irapuato population from Mexico is found in relatively close proximity (100–180 km) to the migratory populations studied here and yet the results shown in Tables 5 and 6 suggest there may be restricted genetic admixture between the two.

It is not clear that microsatellites are the best nuclear markers to address questions of admixture or gene flow. While our microsatellite results are in general agreement with those of Pierce et al. (2014) in suggesting panmixia among migratory and nonmigratory populations in mainland North America (South Florida excluded), the *F*_ST_ comparison between the nonmigratory Irapuato population and the migratory El Rosario population showed borderline significance using a Bonferroni correction (Results). The apparent lack of structure between nonmigratory and migratory populations suggested by microsatellites is in contrast to the evidence for structure from mtDNA (present study) and whole genome analysis (Zhan et al. 2014). Thus, within North America, microsatellites may not be appropriate genetic markers to detect subtle differences in genetic differentiation, although they have proved useful in elucidating migration patterns of monarchs from North America across both the Pacific and Atlantic basins (Pierce et al. 2014). Specifically, potential problems with interpreting microsatellite data in population genetic studies, including evidence from simulation studies that microsatellites often underestimate population differentiation, have been discussed in detail (e.g., Leng and Zhang 2011; Putman and Carbone 2014) and may explain the discrepancies noted here.

Because of the low genetic variability in COI samples of *D. plexippus* from Mexico and Costa Rica analyzed here (haplotype 1 or 2 was present in 97% of the 144 individuals examined in Table 1), clearly the population structure found among migratory and nonmigratory populations based on this gene segment (Table 5) is primarily a result of differences in frequencies of these 2 main haplotypes, with haplotype 1 predominating in migratory populations and haplotype 2 predominating in nonmigratory populations (Figure 1). A search of COI records in GenBank (Appendix Table A1) and BOLD also suggests that this haplotype frequency pattern is robust, although sample sizes currently available are low. Migratory monarchs from Canada (*n* = 6) and USA (*n* = 3) all possessed haplotype 1. Haplotype 1 also is the only haplotype currently recorded in individuals from Australia (*n* = 7) and Spain (*n* = 4), countries which were colonized by migratory monarchs (Zhan et al. 2014). Additional barcode data on monarchs from Costa Rica obtained from phylogenetic tree-based identifications available in BOLD confirmed the predominance of haplotype 2 in nonmigratory individuals. The BOLD trees include sequences not publicly available, but haplotypes can be inferred by comparison with cross-referenced sequences also deposited in GenBank. The BOLD tree indicated that of 56 sequences from Costa Rica corresponding to either haplotype 1 or 2 (which included the 17 GenBank sequences from Figure 1 used as a reference), 46 (82%) were haplotype 2 and only 10 (18%) were haplotype 1. Also, 7 GenBank COI sequences from Costa Rica (574–605 bp), excluded from our genetic diversity analyses because of their shorter length (Appendix Table A1), indicated a low frequency of haplotype 1 (29%) compared to haplotype 2 (57%), and revealed a third individual possessing haplotype 5 (GU333887). The BOLD tree also indicated that haplotype 2 was present in 4 of 5 individuals from Puerto Rico and all 4 individuals from the Dominican Republic, again consistent with the occurrence of high frequencies of haplotype 2 in nonmigratory monarchs from North America.

We also found an apparent shift in the frequencies of the 2 main haplotypes (A and B) of the COII gene segment among migratory monarchs in Mexico (haplotype A predominates) and the nonmigratory Irapuato population (haplotype B predominates) which contributes to the significant population structure observed among the 2 groups (Table 6). Further studies on nonmigratory populations of *D. plexippus* from Mexico, Central America, and South Florida, USA utilizing the mitochondrial COI and COII gene segments, as well as other genetic markers, are needed to provide additional insights into the unresolved issue of the extent of structure and gene flow among the migratory and nonmigratory populations in mainland North America (Pierce et al. 2014; Zhan et al. 2014). This information will be critical in informing future management decisions on conservation and recovery programs of monarchs.

We have included several migratory monarchs from Guaymas, Sonora, Mexico in this study. Coastal Sonora is roughly 1000 km west of the fall migratory route of eastern USA populations of *D. plexippus* to central Mexico (Brower 1995). Monarchs, however, regularly arrive in Sonora in the fall, but are only occasionally seen in the Guaymas area (Pfeiler et al. 2016). Although sample size was small (*n* = 5), data on this population are important because so little is known about migratory corridors in Sonora and possible interactions among monarchs in other populations found farther south. As shown in Figure 1, frequencies of COI haplotypes 1 and 2 are consistent with those of migratory monarchs found in central Mexico. The source of monarchs in Sonora includes migrating individuals from Arizona, which also migrate to overwintering sites in California (Morris et al. 2015) along with most monarchs found west of the Continental Divide (Urquhart and Urquhart 1977). Wild monarchs tagged in Arizona have been found at Bahia de Kino, Sonora (one individual), about 140 km NW of Guaymas, and at the MBBR in central Mexico (15 individuals) (Morris et al. 2015). Important nectar sources for monarchs observed by us in the Guaymas region include coastal sand verbena *Abronia maritima* Nutt. ex S. Watson (Nyctaginaceae), and desert palafox *Palafoxia arida* B.L. Turner & Morris (Asteraceae).

Our findings of a population expansion in *D. plexippus* from the MBBR dating to the late Pleistocene (35000–40000 before present) are in general agreement with results obtained from whole genome analysis which showed a population expansion in monarchs dating to about 20000 years ago (Zhan et al. 2014), although estimates assumed for number of generations per year (3.3) and standard mutation rate (*μ* = 8.4 × 10^–9^) were slightly different than we used. The relatively stable, long-term pre-expansion effective population female size (*N*_ef_) of about 200000 shown in Figure 4 agrees well with the findings of Zhan et al. (2014) (*N*_e_ = about 400000) for the North American population, assuming an equal sex ratio, although estimates of effective population sizes after the expansion differed in the 2 studies. The lack of pre-expansion population fluctuations in the distant past (Figure 4) is routinely seen in Bayesian skyline plots and has been attributed to a loss a signal of ancient population history in contemporary gene sequences (Grant 2015).

The increased availability of milkweed in midwestern USA has been suggested to be the driver of population expansion and migration in monarchs in the late Pleistocene near the end of the last glacial maximum (Zhan et al. 2014). Our results are consistent with the scenario postulated by Zhan et al. (2014) suggesting that migratory monarchs originated in southern USA and northern Mexico, and that nonmigratory populations now present in Central and South America arose from dispersal of this ancestral population. The presence of a rare allele (haplotype 5) found only in Costa Rica (3 individuals) is consistent with a founder event scenario in which initially rare alleles in source populations increase in frequency by “genetic surfing” during population expansions (Edmonds et al. 2004; Slatkin and Excoffier 2012).

Reliable estimates of effective population size (*N*_e_), which are typically lower than census population sizes, are important parameters in conservation biology in that they provide a means to evaluate threats to the genetic health of populations (Hare et al. 2011). Also, uncertainties and controversies over methods and assumptions currently used in estimating census population size in migrating monarchs, both in the overwintering sites and summer ranges (Dyer and Forister 2016; Pleasants et al. 2016), emphasize the need for obtaining estimates of *N*_e_ utilizing a variety of methods (Hare et al. 2011). A recent decline of 90% in the overwintering monarch population in the MBBR, indicated by habitat loss and census approaches (Davis and Rendón-Salinas 2010; Vidal and Rendón-Salinas 2014) can be examined using genetic approaches, but would require a comparison of samples taken over multiple years. Such monitoring, using molecular methods, would reveal not only possible changes in *N*_e_, but in the potential loss of the genetic diversity so critical to conservation management and planning.

## Appendix

**Table:** **Table A1**. GenBank records analyzed for the standard COI barcode segment of adult *Danaus plexippus*, with haplotype designations used in the present study

Country	GenBank accession no.	Voucher code	Date collected	COI haplotype	Base pairs (bp)
Costa Rica^a^	GU333882	01–SRNP–25029	17 Dec 2001	2	574
	GU333883	00–SRNP–20751	20 Nov 2000	2	574
	GU333885	98–SRNP–7209	19 Aug 1998	2	574
	GU333886	98–SRNP–7254	24 Aug 1998	2	605
	GU333887	00–SRNP–353	25 Feb 2000	5	591
	GU333888	98–SRNP–7210	20 Aug 1998	1	572
	GU333889	00–SRNP–353	16 Feb 2000	5	548
	GU651737	09–SRNP–495	25 Feb 2009	2	658
	GU666763	08–SRNP–70857	07 Jul 2008	2	658
	GU666764	08–SRNP–31170	26 May 2008	2	658
	GU666765	08–SRNP–70296	19 May 2008	2	658
	GU666766	08–SRNP–70297	21 May 2008	2	658
	GU666769	08–SRNP–72358	24 Sep 2008	2	658
	GU666770	08–SRNP–12361	19 Aug 2008	2	658
	HM884512	10–SRNP–103587	02 Apr 2010	2	658
	HM905228	10–SRNP–103418	27 Mar 2010	1	658
	HM905373	10–SRNP–103569	01 Apr 2010	1	658
	HM905374	10–SRNP–103570	01 Apr 2010	2	658
	JQ529767	10–SRNP–67640	05 Jul 2010	5	658
	JQ529768	10–SRNP–67641	06 Jul 2010	5	658
	JQ529799	10–SRNP–67008	27 Jan 2010	2	658
	JQ543430	06–SRNP–3790	20 May 2006	1	656
	JQ548240	05–SRNP–43782	05 Jan 2006	2	658
	JQ536356	07–SRNP–45296	09 Jun 2007	2	658
	JQ537098	07–SRNP–65886	15 Nov 2007	2	657
	JQ553760	08–SRNP–108642	28 Nov 2008	2	658
Yucatan Peninsula, Mexico	GU659705	MAL–02630	04 Jul 2002	1	588
	GU659706	MAL–02631	05 Jul 2002	2	644
	GU659707^b^	MAL–02633	24 May 1999	2	621
	GU659709	MAL–02626	08 Sep 2006	2	658
	GU659710	MAL–02627	07 Nov 2003	1	658
	GU659711	MAL–02628	11 Mar 2004	6	658
	HM890805	MAL–02629	25 Nov 2004	2	549
Canada (Ontario)	KM550302	PPBP-1165	12 Jun 2008	1	658
Australia	KF398175	11ANIC-06932	02 Feb 1968	1	658
	KF402604	11ANIC-06933	01 Dec 1984	1	658
	KF403674	11ANIC-06931	23 Mar 1996	1	658
Spain	GU675768	SMcoll.08-J600	22 Jun 2008	1	658
	JN266597	RVcoll.130209KL61	01 Dec 2001	1	658
	KP870908	RVcoll. 07-C577	20 Jul 2005	1	658
	KP871034	RVcoll. 07-C576	20 Jul 2005	1	658

All sequences, except the one from Canada, are from nonmigratory populations.

a Most adult monarchs from Costa Rica were obtained from larvae reared in the laboratory on *Asclepias curassavica* L. (Apocynaceae) (Janzen and Hallwachs 2009).

b Examination of the original electropherogram revealed 3 sites in the 621 bp segment (sites no. 88, 187, and 190) that contained reading errors in the output file which were corrected.
